# Identifying clusters of leprosy patients in India: A comparison of methods

**DOI:** 10.1371/journal.pntd.0010972

**Published:** 2022-12-16

**Authors:** Anneke T. Taal, Akshat Garg, Suchitra Lisam, Ashok Agarwal, Josafá G. Barreto, Wim H. van Brakel, Jan Hendrik Richardus, David J. Blok

**Affiliations:** 1 NLR, Amsterdam, The Netherlands; 2 Erasmus MC, University Medical Center Rotterdam, Rotterdam, The Netherlands; 3 NLR India, Delhi, India; 4 Federal University of Pará, Belém, Brazil; The University of Hong Kong, CHINA

## Abstract

**Background:**

Preventive interventions with post-exposure prophylaxis (PEP) are needed in leprosy high-endemic areas to interrupt the transmission of *Mycobacterium leprae*. Program managers intend to use Geographic Information Systems (GIS) to target preventive interventions considering efficient use of public health resources. Statistical GIS analyses are commonly used to identify clusters of disease without accounting for the local context. Therefore, we propose a contextualized spatial approach that includes expert consultation to identify clusters and compare it with a standard statistical approach.

**Methodology/Principal findings:**

We included all leprosy patients registered from 2014 to 2020 at the Health Centers in Fatehpur and Chandauli districts, Uttar Pradesh State, India (n = 3,855). Our contextualized spatial approach included expert consultation determining criteria and definition for the identification of clusters using Density Based Spatial Clustering Algorithm with Noise, followed by creating cluster maps considering natural boundaries and the local context. We compared this approach with the commonly used Anselin Local Moran’s I statistic to identify high-risk villages. In the contextualized approach, 374 clusters were identified in Chandauli and 512 in Fatehpur. In total, 75% and 57% of all cases were captured by the identified clusters in Chandauli and Fatehpur, respectively. If 100 individuals per case were targeted for PEP, 33% and 11% of the total cluster population would receive PEP, respectively. In the statistical approach, more clusters in Chandauli and fewer clusters in Fatehpur (508 and 193) and lower proportions of cases in clusters (66% and 43%) were identified, and lower proportions of population targeted for PEP was calculated compared to the contextualized approach (11% and 11%).

**Conclusion:**

A contextualized spatial approach could identify clusters in high-endemic districts more precisely than a standard statistical approach. Therefore, it can be a useful alternative to detect preventive intervention targets in high-endemic areas.

## Author summary

Leprosy is chronic infectious disease characterized by skin and peripheral nerve lesions. Despite the efforts to eliminate leprosy, around 210,000 new cases are still found annually of which 60% is reported by India alone. To reduce the incidence significantly, new active case finding approaches in combination with preventive treatment to at-risk populations are needed in high-endemic areas in India. Geospatial methods can support program managers and policy makers to identify clusters of leprosy patients and target at-risk populations for preventive interventions. However, often standard spatial methods do not account sufficiently for the local context (i.e., local barriers and social determinants). In this study, we describe a contextualized spatial approach that includes expert consultation to identify context specific clusters and compared it with a standard approach. Overall, our results show that the contextualized approach is able to identify more clusters precisely and covers a larger proportion of the population in clusters that would need to be targeted for preventive interventions. For program managers and policy makers, the contextualized approach can be useful to target at-risk populations in high-endemic areas while ensuring efficient use of public health resources. Further research is needed to test the scalability in different endemic settings and to apply to other Neglected Tropical Diseases.

## Introduction

Leprosy is caused by an infection with *Mycobacterium leprae*. It affects the peripheral nerves and skin, and if left untreated, can progress to lifelong disabilities [1,2]. Therefore, patients affected by leprosy, and especially those who develop disabilities, often suffer from social stigma and discrimination leading to social exclusion, economic loss, and depression [3]. The World Health Organization (WHO) still reports 200,000 new cases of leprosy annually, of which 80% are registered in India, Brazil, and Indonesia [4].

India alone reported 114,451 new cases in 2019, which are unevenly distributed within the country. In many districts, high endemic pockets with ongoing transmission exist [4]. Despite the many efforts to eliminate leprosy, new cases are still found in India during past and current leprosy case detection campaigns [5,6]. Therefore, new active case finding approaches in combination with prophylactic treatment to at-risk populations are needed to reduce the incidence considerably in these high endemic areas in India [7,8].

Progress have been made in the field of prophylactic interventions to prevent leprosy among at risk populations [9]. The COLEP study demonstrated that a single dose of rifampicin provided as post-exposure prophylaxis (SDR-PEP) to contacts of leprosy patients reduces the risk of developing leprosy by, on average, 57% [10]. SDR-PEP has now been taken up in the WHO leprosy guidelines to be used as preventive treatment for contacts of leprosy patients after excluding leprosy and tuberculosis disease [11]. Recently, new studies have started to assess the effectiveness of novel PEP strategies and/or PEP regimens. For example, the PEP++ trial compares the effectiveness of an enhanced PEP regimen, a combination of two antibiotics, with SDR-PEP administered to close contacts of leprosy patients in high-endemic areas in five countries. In this trial, enhanced PEP is provided in combination with a cluster-based door-to-door campaign. People within identified clusters will be screened for leprosy signs and symptoms and provided with PEP.

Geographic Information System (GIS) or spatial analysis are frequently used to identify clusters of disease and to measure spatial patterns that are not random. Among these clustering tools, many statistical methods have been developed to verify if disease clusters are of sufficient geographic size to have not occurred by chance [12]. Also in leprosy, spatial techniques are perceived as an important tool to identify clusters [13,14]. Global clustering methods such as the Global Moran’s I statistic are used to indicate the occurrence of spatial clustering in the complete area and are often followed by local clustering methods [14,15]. Local clustering methods such as Local Moran’s I statistic (Local Index of Spatial Association [LISA]) and Getis Ord Gi* statistic are commonly used to identify areas of significant high leprosy incidence and to find correlations with high grade 2 disability 2 (G2D) rates and low socioeconomic status [15–19]. With Kulldorff’s Spatial and Space Time Scan Statistics (SaTScan) [20], a space-time local clustering technique that can adjust for heterogeneous background population densities and confounding variables [21], the most likely cluster(s) of disease are identified controlled for gender, age, type of leprosy and deformity, and over a period of time [22–24]. Non-statistical spatial methods such as the Kernel Density Estimation which estimates the probability density function of a random variable, are applied to visualize clusters or so-called hotspots of disease and disability [14,25,26].

Statistical spatial methods are mainly used to test a hypothesis and identify clusters in leprosy research context. These methods can control for possible confounding and adjust for population densities, but do not account for the local barriers or social determinants. Involvement of local communities and stakeholders for identifying high risk areas (intervention target) is beneficial as they provide valuable information on the local context such as the natural boundaries, presence of stigma or travel and social behavior of the population in a specific area [27]. Program managers and policy makers intend to use spatial tools to identify the at-risk populations for preventive interventions while considering efficient use of public health resources [13,28]. They often rely on simple maps that present the leprosy incidence, grade 2 disability or child rate per district only. Therefore, to guide program managers and policy makers for identification of at-risk clusters, a contextualized spatial approach that allows them to identify clusters while considering the local context would be valuable.

In the PEP++ trial, a contextualized spatial approach was developed to identify high-risk areas for door-to-door PEP interventions, and not necessarily to find statistical clusters. This approach includes GIS spatial methods and expert consultation to account for the local context. Non-statistical methods were required to identify clustering at a local level using individual level data (i.e., location of the house of the leprosy patient) and can include considering of local barriers. This study describes the contextualized spatial approach that has been used in the PEP++ trial to identify clusters of leprosy patients targeted for active case finding and preventive intervention and compares it with a standard statistical spatial analysis method.

## Methods

### Ethics statement

The PEP++ trial received ethical clearance from the Vardhman Mahavir Medical College & Safdarjung Hospital (IEC/VMMC/SJH/Project/2019-09/40) and is approved by the Indian Council of Medical Research (ICMR) in New Delhi, India on September 17, 2018. For this study, ethical clearance has been obtained for collecting patient information from the national register and the GPS coordinates of patients registered from 2014 to 2020 at the public health centers in Chandauli and Fatehpur. A copy of the dataset has been anonymized to perform the analysis.

### Study area

**Fig 1:**
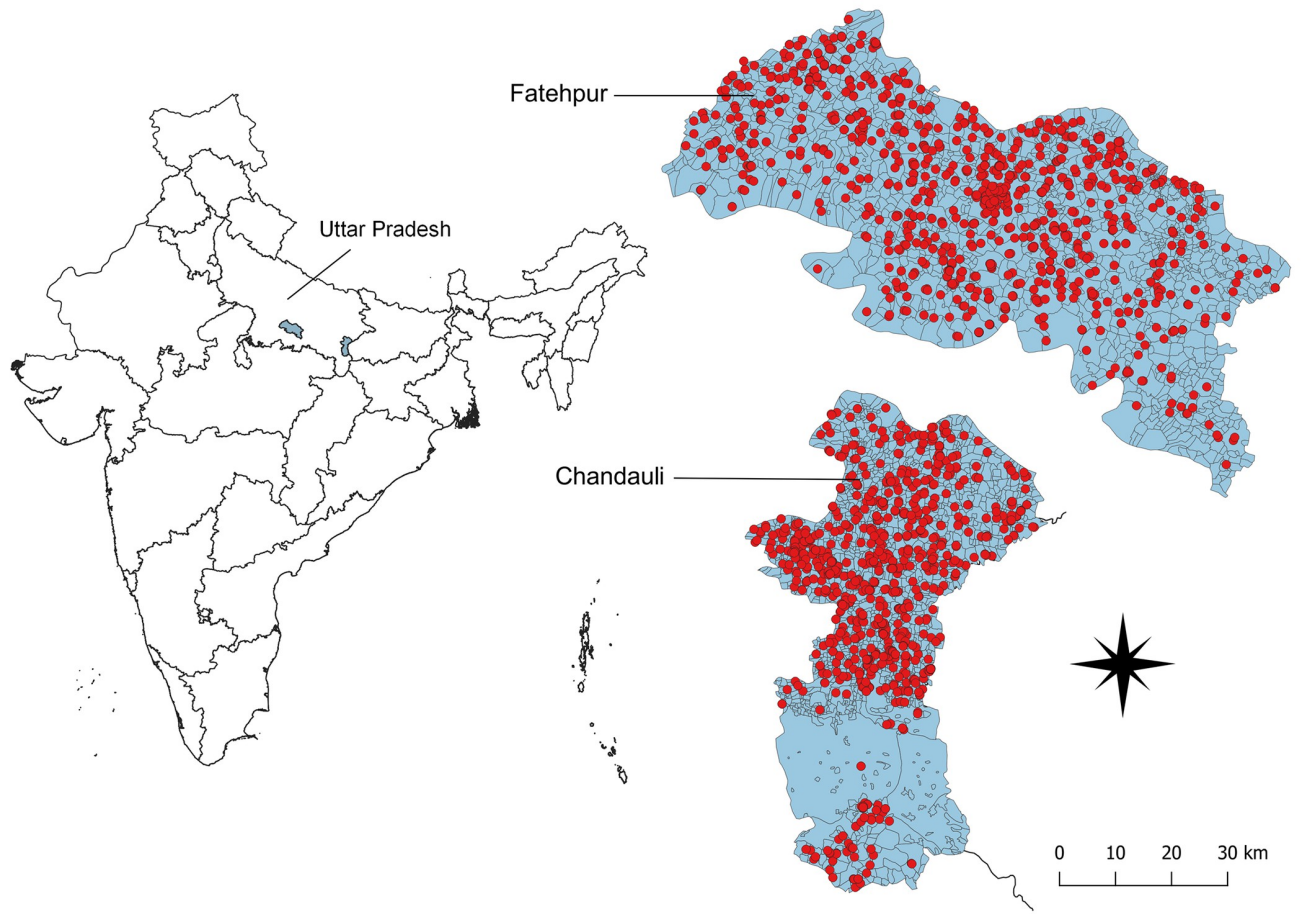
Overview of the study area in Uttar Pradesh, India, which covers Fatehpur and Chandauli district. The red dots represent the distribution of the mapped leprosy index cases registered from 2014 to 2020. Base layers from https://sedac.ciesin.columbia.edu/data/set/india-india-village-level-geospatial-socio-econ-1991-2001/data-download.

This study used spatial and demographic data of registered leprosy patients in Chandauli and Fatehpur districts, Uttar Pradesh (UP) State, India. UP is one of the most endemic states of India with an annual new case detection rate (ANCDR) of 65.8 per 1,000,000 population in 2019 [29]. Chandauli district is in southeast of UP and borders Bihar State. It has an area of 2,484 km^2^ and population of 2.34 million in 2020 [Table B in S1 Text]. Fatehpur district is located in central south of UP and has an area of 4,152 km^2^ and population of around 3.10 million in 2020 [Table B in S1 Text]. Both districts are considered high endemic for leprosy with an ANCDR of 54.6 per 1,000,000 population in Chandauli and of 76.6 per 1,000,000 population in Fatehpur in 2021 [Table B in S1 Text]. Fig 1 shows the geographic location of the two districts.

### Data collection

We collected the medical records of all leprosy index cases registered from April 2014 to March 2020 across 10 Primary Health Centers (PHCs) and 17 PHCs in Chandauli and Fatehpur, respectively. Between January 2018 and June 2020, we conducted five mapping surveys to collect the spatial data based on information on patient’ names and addresses with village name. Project staff were trained in data collection using the mobile application MapIt software (version 7.6.0, https://mapitgis.com/), a tool for Geographic Positioning Systems (GPS) data collection and management, in January 2018. They were assigned to one of the blocks and worked together with Indian government staff, such as Medical Officers (MOs), Non-Medical Assistants (NMAs), Paramedical workers (PMWs) of the PHC and Accredited Social Health Activists (ASHAs) as community volunteers. Since we only had the village and patient name, the support of PHC staff and ASHAs including the chief of village (i.e., *pradhans*) was required to find the patients’ houses. Once a patient’s house was located, the GPS coordinates of the house were recorded offline with MapIt. All data points were uploaded to the server in the NLR India office in New Delhi to be aggregated and combined with demographic information for analysis. Table A in S1 Text shows an overview of the five mapping surveys. All data were imported in the open-source Quantum Geographic Information System (QGIS) version 3.4.1 (QGIS Developer team, Madeira 2018)) for validation. Incorrect data points (e.g., situated in a lake or field) were removed and re-collected by project staff.

### Spatial analysis: Contextualized spatial approach

**Fig 2:**
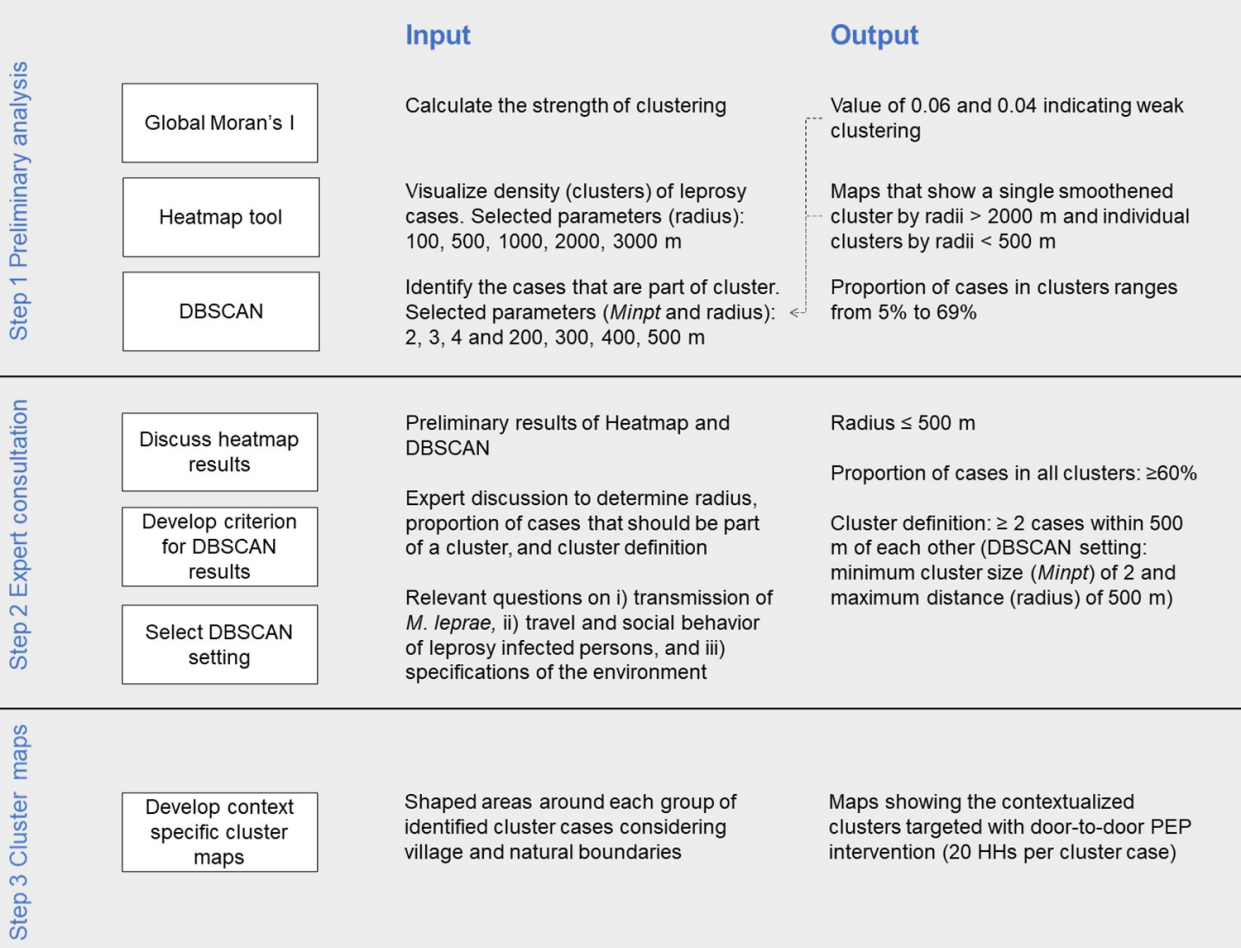
Overview of the different steps in the contextualized spatial approach with the input and output by step. Step 1 is the preliminary analysis using the Global Moran’s I, Heatmap and the DBSCAN tool with the data of 2014–2017. The output of the Global Moran’s I and Heatmap are used for the selection of parameters of the DBSCAN (arrows). Step 2 is the expert consultation during which the preliminary results are discussed followed by decisions that have been taken as criteria on cluster definition. Step 3 is the development of context specific cluster maps for the PEP interventions. Minpt: minimum number of points in a cluster; PEP: post-exposure prophylaxis; HH: household.

We developed a contextualized spatial approach for the PEP++ trial to identify clusters of leprosy cases that account for the local barriers and social determinants. It comprises of the non-statistical Density-Based Spatial Clustering of Applications with Noise (DBSCAN) tool and an expert consultation to identify clusters of leprosy cases (high-transmission areas) at individual level. The approach was divided into three steps: a preliminary spatial analysis, an expert consultation to decide on criteria and a cluster definition, and the development of context specific cluster maps that can be used in the field. Fig 2 shows an overview of the steps, and S1 Text describes the complete methodology used in this approach.

#### Step 1 Preliminary spatial analysis

In the preliminary analysis, we analyzed the data on strength of clustering, then visualized clusters, and finally identified cases that were part of each main cluster. The strength of clustering of leprosy patients was calculated using Global Moran’s Index statistic [30] in GeoDa (https://geodacenter.github.io/) version 1.18 (Anselin, Santa Barbara, CA, USA). It calculates the autocorrelation coefficient (degree of similarity) between spatial points (and takes values ranging between -1 and 1), where -1 indicates dispersed distribution, 0 no clustering, and 1 strong clustering. In this approach, the Global Moran’s I was used to determine reasonable cluster sizes. Strong clustering indicates that spatial points are located close together and therefore a cluster can contain many patients. Weak clustering indicates that only a few spatial points are close together and therefore a cluster should contain a few patients.

Clustering of cases was visualized using the heatmap tool (Heatmap plugin, QGIS). The heatmap tool draws a circle with a specified radius (e.g., 1000 m) around each data point and creates a raster file as output. Then, it uses Kernel Density Estimation to calculate the density of points for each raster cell. Raster cells that are close to data points will have a higher density (higher value) compared to raster cells that are further away which will have a low density (low value). The resulting map showed the density distribution of clusters and non-clusters. In this approach, the heatmap radius was used to select the maximum distance between patients that would be part of a cluster. A heatmap that shows many individual density spots can be selected as the maximum distance.

To identify cases that are part of a cluster, we used the DBSCAN tool [31] of QGIS. This tool focuses on the proximity and density of points to form (arbitrary shaped) clusters. A minimum cluster size (minimum points; *MinPts*) and maximum distance to a nearest neighbor (radius; ε) needs to be selected beforehand. In the DBSCAN tool, points can be classified as core points, reachable points and outliers. A point is identified as a core point if it has the specified minimum number of points in its radius-neighborhood (including the core point). A reachable point is a point within the radius-neighborhood of the core point and is identified as part of the cluster. All points outside the radius-neighborhood are ‘outliers’ or ‘noise points’ and will not be identified as cluster points. The parameters of the DBSCAN were determined by the strength of clustering (Global Moran’s I) and density of points (Heatmap). We used the DBSCAN tool with 12 different combinations of minimum cluster size (i.e., 2, 3, 4) and maximum distance (i.e., 200 m, 300 m, 400 m, and 500 m). For each combination, we calculated the proportion of the total cases georeferenced that would be part of a cluster.

#### Step 2 Expert consultation

An expert consultation was organized with the aim to i) discuss the preliminary results of the clustering analysis, ii) decide which cluster definition to use in the concerned setting and iii) discuss the implications of this choice for the door-to-door campaigns. Among the participants were representatives of the state and district government health department, health care workers of both districts, and project staff that collected the data (n = 60). Their knowledge and experience of the Chandauli and Fatehpur context was important to determine the definition of a cluster.

To get a better understanding of the Chandauli and Fatehpur context, additional information about i) transmission of *M*. *leprae* in the two districts, including MB leprosy, child, and grade 2 disability proportions, ii) the travel and social behavior of leprosy infected persons, and iii) specifications of the environment (i.e., districts), including rural or urban areas, natural and social boundaries, population counts, poverty, presence of violence or stigma and access to health centers, were gathered and discussed.

The experts recommended using: a small cluster size (*Minpt*) and small maximum distance (ε, radius) for the cluster analysis and that ‘at least 60% of the leprosy cases should be part of a detected cluster’ (S1 Text). Based on this input the appropriate DBSCAN parameter combination was selected, which included a minimum cluster size of 2 and maximum distance of 500 m (i.e., at least 2 cases living within 500 m of each other; Table C in S1 Text).

#### Step 3 Develop context specific cluster maps

The data points that are within 500 m of another point were identified as a cluster case and used for further analysis. Cases that are part of the same cluster were grouped and we drew a polygon shape around them using Google Satellite Imagery. While shaping the areas, we considered the village, street and natural boundaries, and capturing on average 20 households (i.e., 100 individuals) around each cluster case as was decided during the expert consultation.

### Spatial analysis: Statistical approach

Statistical tools are often used by academics to identify significant clusters with risk ratios. We used the Local Moran’s Index (Local Indicator of Spatial Association [LISA]) [32] because it is a commonly used statistical method to test for local clustering and can be used to identify the highest risk areas of occurrence of leprosy in Fatehpur and Chandauli district. As the areas of interest, we selected village level since this is the smallest area with known population data [33,34].

Similar as the Global Moran’s I, the Local Moran’s Index measures the correlation coefficient (degree of similarity) between neighbouring spatial points. The difference is that the Global Moran’s I calculates one summarizing I value for the complete study area and the Local Moran’s calculates the I value for each spatial point (local level). In this study, we calculated the correlation coefficient between villages with or without leprosy cases to identify clustering of villages. The algorithm removes the village from its neighborhood and determines if the neighborhood is significantly different from the study area, then it determines if each village is significantly different from its neighborhood. The tool calculates the Moran’s Index value and both a z-score and p-value to evaluate the significance. A Moran’s I value of zero indicates homogenous distribution (no clustering). A value between 0 and 1 indicates that a village has neighboring villages with similarly high or low number of leprosy cases; this village is part of a cluster. A value between -1 and 0 indicates that a village has neighboring villages with dissimilar number of cases in the village; this village is an outlier.

We checked the data for outliers using the histogram function in GeoDa version 1.18. Since the data showed a non-normal distribution with extreme high values, we decided to use the ‘Univariate Median Local Moran’s I’ tool in GeoDa to identify the high-risk cluster villages. This is an extension of the Univariate Local Moran’s I tool that can correct for the variance caused by extreme high or low values. We appointed the total number of cases per village as ‘event’ variable. The queen contiguity with first order was selected to determine the neighbors, which includes all neighboring villages that directly share a spatial border with the village of interest. To construct the reference distribution, we selected the Monte Carlo replication of data sets of 999 permutations (i.e., pseudo p value of 0.001) to ensure adequate power for defining clusters. As the output, GeoDa provides a significance map and a cluster map. The significance map shows the villages with a significant local I statistic (p value ≤ 0.05). The thematic cluster map shows the clusters high-high (i.e., village with high number of cases surrounded by villages with high number of cases) and low-low (i.e., village with low number of cases surrounded by villages with low number of cases), the outliers high-low (i.e., village with high number of cases surrounded by villages with low number of cases) and low-high (i.e., village with low number of cases surrounded by villages with high number of cases), and the villages that are not significant. In this study, the high-high and high-low villages (villages with leprosy cases and a p value lower than 0.05) were considered as leprosy cluster.

### Outcome measures

To compare the contextualized spatial and statistical approach, the proportion of cases in clusters, the number of clusters, the cluster area, the total number of people in clusters, and the proportion of contextualized clusters that overlap with statistical clusters are calculated for urban and rural areas in both districts and for each approach. In the contextualized spatial approach, the proportion of cases in clusters was calculated as the number of leprosy cases in clusters divided by the total number of leprosy cases. The total population in clusters is the sum of populations as provided by the pradhans of the villages with leprosy clusters in 2021. In the statistical approach, the proportion of cases in clusters was calculated as the number of leprosy cases in high-high and high-low villages divided by the total number of leprosy cases. The total population in clusters is the sum of the populations in high-high and high-low villages. The proportion of cluster area that overlap will be calculated as the total area overlap divided by the total cluster area of the contextualized spatial approach.

The implications for the door-to-door active case finding and PEP interventions in the clusters were also compared for the two approaches. The estimated number of people to be targeted with PEP was calculated for both approaches as the number of cases in clusters multiplied by 100 individuals. Following the PEP++ strategy, we assumed that 20 households which is about 100 individuals were targeted per leprosy case in a detected cluster. The proportion of total population in the clusters to be targeted for PEP was also calculated for both approaches.

## Results

### Distribution of cases

From April 2014 to March 2020, in total, 4,039 new leprosy cases were registered at the PHCs, 1,710 in Chandauli district and 2,329 in Fatehpur district. The locations of 1,647 (96.3%) and 2,208 (94.8%) leprosy patient’ houses were georeferenced during the five mapping studies in Chandauli and Fatehpur, respectively. The New Case Detection Rate (NCDR) per village was calculated as the total number of leprosy cases registered from April 2014 to March 2020 in the village divided by the population of the village in 2021. Fig A in S1 Text shows the NCDR per 1,000,000 population for Chandauli and Fatehpur. A Moran’s I statistic of 0.030 (z score of 2.078; p value of 0.031) and 0.028 (z score of 2.624; p value of 0.018) in Chandauli and Fatehpur respectively, indicates weak clustering of cases. The heatmaps of the two districts showed many medium density areas and a few hotspots of leprosy cases (Fig B in S1 Text).

### Comparison of the contextualized spatial approach with standard statistical approach

**Fig 3:**
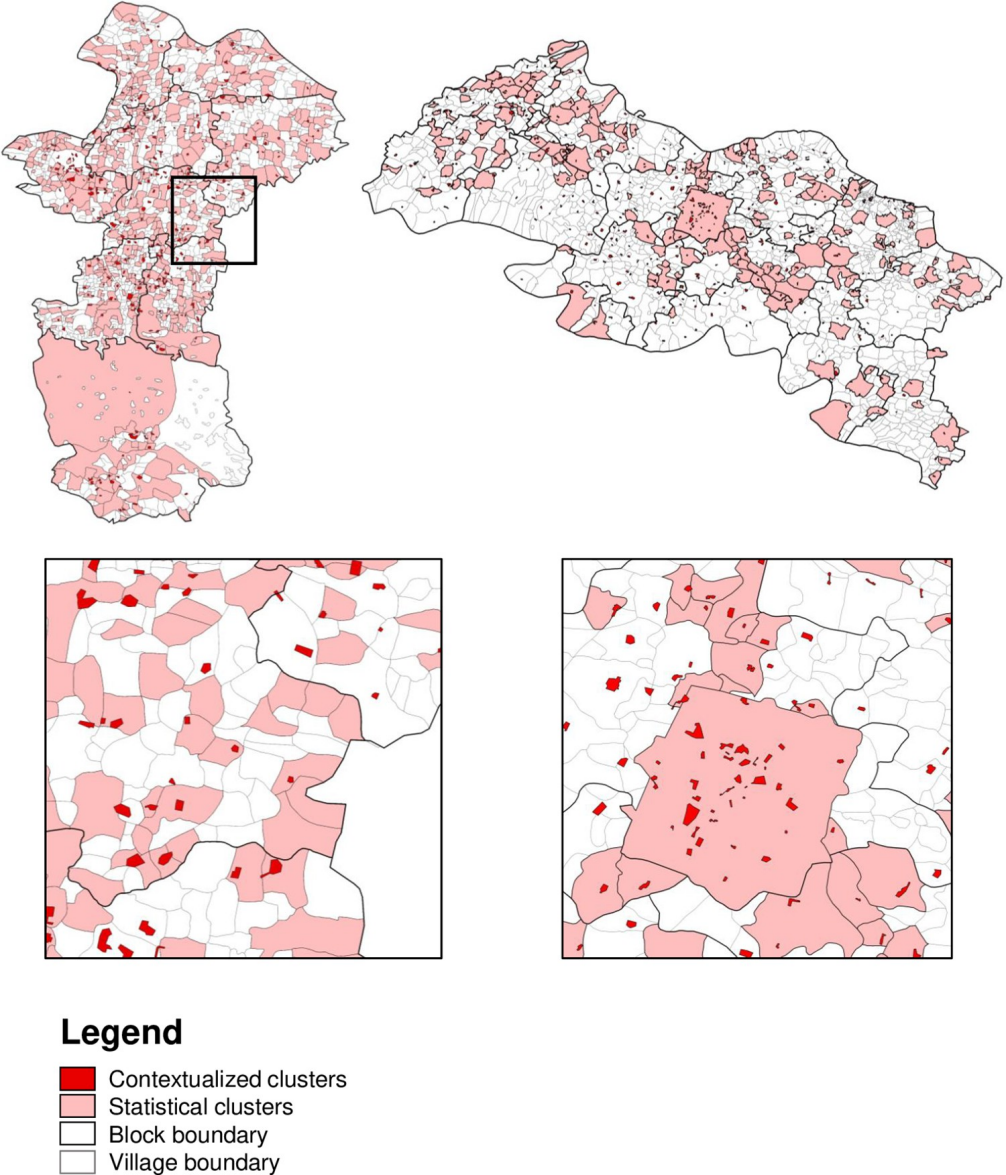
The contextualized clusters and the statistical cluster villages in Chandauli district (top left) and Fatehpur district (top right), and a zoom-in of clustering in a rural area in Chandauli (bottom left) and in an urban area in Fatehpur (bottom right). The bright red areas are the contextualized clusters identified in the contextualized spatial approach and the pink areas are the significant statistical cluster villages identified by Local Moran’s I. The zoom-in of the rural area shows that both approaches are able to identify clusters. Base layers from https://sedac.ciesin.columbia.edu/data/set/india-india-village-level-geospatial-socio-econ-1991-2001/data-download.

Fig 3 shows the contextualized clusters identified by the contextualized spatial approach (dark red) and the cluster villages identified by the statistical approach (light pink). In the contextualized spatial approach, 374 clusters are identified in Chandauli and 512 cluster in Fatehpur. These clusters are distributed throughout the districts. In the statistical approach, 508 cluster villages are identified in Chandauli and 193 cluster villages in Fatehpur. The cluster villages in Chandauli are distributed throughout the district and the cluster villages in Fatehpur, are identified in the center and North part of the district.

The results and the implications for PEP interventions of both approaches for Chandauli and Fatehpur are compared and presented in Table 1 and Table 2. Overall, the indicators were lower for urban compared to rural clusters and villages. The proportion of cases in clusters for urban and rural areas together is 75.0% and 57.2% in Chandauli and Fatehpur, respectively, in the contextualized spatial approach, and 66.2% and 43.0% in the statistical approach (Table 1). The proportion of total population in clusters that would be targeted for PEP (20 households per leprosy cluster case) is 31.3% in Chandauli and 11.4% in Fatehpur in the contextualized spatial approach, and 10.8% and 10.8%, respectively in the statistical approach (Table 2). The proportion of contextualized clusters that overlap with statistical clusters is for Chandauli 41.3% and 62.0% in urban and rural areas, respectively, and for Fatehpur, this is 60.6% and 39.8% (Table 1).

**Table 1:** The results of two approaches compared.

	Contextualized spatial approach						Standard statistical approach					
	Chandauli*			Fatehpur*			Chandauli			Fatehpur		
	Urban	Rural	Total	Urban	Rural	Total	Urban	Rural	Total	Urban	Rural	Total
Proportion of cases in clusters^a^	7.2%	67.8%	75.0%	12.7%	44.5%	57.2%	3.0%	62.1%	65.1%	13.1%	29.9%	43.0%
Number of clusters	44	330	374	123	389	512	10	498	508	23	170	193
Cluster area km^2^	3.1	32.3	35.4	7.8	25.8	33.7	32.9	1,236.2	1,269.1	377.0	569.3	946.3
The total population in clusters^b^	60,000	335,000	395,000	276,000	834,000	1,110,000	132,000	859,000	991,000	463,000	413,000	876,000
Proportion cluster area overlap^c^	41.3%	62.0%	60.3%	60.6%	39.8%	44.5%						

* Chandauli has 20 urban areas (total population of 264,000) and 1405 rural areas (total population of 1,939,000), Fatehpur has 56 urban areas (total population of 356,4,000) and 1296 rural areas (total population of 2,641,000) [33]

a Number of cluster cases divided by total number of cases for the contextualized spatial approach and the number of cases in red and pink villages divided by the total number of cases for the statistical approach

b Calculated as the sum of populations in each cluster as estimated by the head of village in the contextualized spatial approach and as the sum of populations in cluster villages for the statistical approach [33]

c Calculated as the total cluster area that overlap with each other divided by the total cluster area of the contextualized spatial approach.

**Table 2:** The implications for PEP interventions of two approaches compared.

	Contextualized spatial approach						Standard statistical approach					
	Chandauli			Fatehpur			Chandauli			Fatehpur		
	Urban	Rural	Total	Urban	Rural	Total	Urban	Rural	Total	Urban	Rural	Total
Estimated population targeted for PEP intervention^a^	11,900	111,700	123,600	28,100	98,300	126,400	5,000	102,300	107,300	29,000	66,000	95,000
Proportion of total population in clusters targeted for PEP^b^	19.8%	33.3%	31.3%	10.2%	11.8%	11.4%	3.8%	11.9%	10.8%	6.3%	16.0%	10.8%

a Calculated as the number of cluster cases multiplied by 100 individuals (20 households)

b Calculated as the estimated population targeted for PEP intervention divided by total population in clusters

## Discussion

Our study described a contextualized spatial approach with expert consultation to identify contextualized clusters of leprosy cases to target door-to-door PEP interventions in two high-endemic districts in India and compared this approach with a standard statistical approach. Both approaches could identify clusters of cases in the two high-endemic districts. The contextualized spatial approach, however, identified smaller clusters and a higher proportion of cases in clusters in both districts. This may imply that the contextualized approach identified clusters more precisely in high-endemic districts. Also, the total population in clusters targeted for PEP were smaller in urban settings of both districts in the contextualized approach compared to the statistical approach.

As program managers often operate with limited budgets and resources, it is necessary to target PEP interventions only to those that are most likely to be infected with *M*. *leprae* and are at risk of developing leprosy. Our findings showed that the contextualized spatial approach covers a higher proportion of cases in clusters of rural and urban areas combined and therefore, results in larger number of individuals targeted for PEP intervention. A contextualized spatial approach is very suitable if one aims to target the complete cluster or target the at-risk population in smaller but more precise clusters that account for the local context. Especially with door-to-door campaigns in urban areas, targeting smaller and more precise clusters within a city can be more efficient in terms of resources and time compared to large urban clusters as identified in the statistical approach. The statistical spatial approach should be considered if the local context is not considered relevant and if targeting larger clusters is acceptable, or if the interest is to target only the clusters that are identified as statistical significant (i.e., areas where cases are not appearing randomly). Generally, this approach would result in fewer and larger urban clusters. Moreover, the proportion of total cases in clusters (urban and rural) is lower resulting in a lower number of individuals targeted for the PEP intervention. This implies that a (significant) part of the at-risk population may be missed for preventive interventions.

Additional considerations for choosing a suitable and feasible spatial approach for a particular district could be: i) population density, ii) granularity of geographic data, and iii) time and resources available for the data collection and analysis. Our contextualized spatial approach can be applied in areas with both low and high population densities, where preferably individual level data is available. However, in our study this approach required at least four weeks to collect the data, including, GPS coordinates of patients and information about local barriers, epidemiological indicators, social determinants, presence of stigma, migration and an estimation of the population, and another two weeks for the data manager to construct the context specific maps. Door-to-door campaigns (with PEP) would therefore require additional time and resources for planning but may save resources (precise clusters and less travelling) for the actual implementation. Nevertheless, this approach would only be feasible when enough time and resources are available to carry this out. Otherwise, we recommend using a standard statistical approach. A statistical approach is also recommended if the population is distributed heterogeneously and if data is only available at village or health center level.

The expert consultation in our study was the added value of the contextualized approach. Expert consultations or key stakeholder meetings are often used in leprosy to gain support for new ideas or strategies. For example, at global level to develop leprosy elimination strategies or guidelines for treatment [35], at national level to improve leprosy control activities [36], or at research level to develop innovative tools or a research agenda [37,38]. The success of our expert consultation and development of context specific cluster maps depended on relevant questions related to the i) transmission of *M*. *leprae* in the two districts, including MB leprosy, child, and grade 2 disability proportions, ii) the travel and social behavior of leprosy infected persons, and iii) specifications of the environment (i.e., districts), including rural or urban areas, natural and social boundaries, population counts, poverty, presence of violence or stigma and access to health centers. This resulted in an understanding of the clustering of leprosy cases, a cluster definition and methodology based on the travel behavior of population in the concerned setting, context specific cluster maps presenting more precise target areas for PEP, and a PEP door-to-door strategy considering available resources in the two districts.

The transmission of *M*. *leprae* and clustering of cases can be different in rural and urban areas as a result of migration to cities, higher awareness among urban population, and closer distance to an infected person [39,40]. Mohite *et al*. found an increase in number of child cases in urban areas compared to rural areas indicating that urban areas become an important source of transmission and therefore, may need a different clustering approach and possible PEP strategy [41]. Currently, urban clusters are mainly identified using statistical tests, including Kulldorff’s spatial scan statistic [42,43]. In our study, we did not use a different spatial approach for urban or rural areas. However, we observed that the contextualized spatial approach is able to identify more precise and smaller clusters in urban areas than the statistical spatial approach, resulting in a higher proportion of total population in clusters targeted for PEP. An example is Fatehpur city (Fig 3, zoom in). The city is identified as a cluster village by the statistical spatial approach and therefore, for each leprosy case in the city 100 individuals will be targeted for door-to-door PEP interventions. In the contextualized spatial approach, we could identify small individual clusters that are mainly located in the center of Fatehpur. This implies that more transmission is taking place in the center than at the outskirts of the city. As a result, the leprosy cases that are not within a distance of 500 m from another case would not be targeted for door-to-door PEP interventions and therefore, the proportion of total population in clusters targeted for PEP is higher compared to the statistical approach. Considering more efficient use of public health resources during active case finding and PEP intervention and limiting exposure to stigma, our contextualized spatial approach could be very useful to identify more precise clusters, especially in urban areas.

The maximum proportion of cluster overlap was 60% indicating that the two approaches can identify the same clusters but also have 40% of the clusters not in common. In clusters identified by the statistical approach only, leprosy cases were located more than 500 meters from each other, and therefore not identified as a cluster in the contextualized approach. In clusters identified by our contextualized approach only, the number of leprosy cases in a village was considerably lower (or equally low) compared to neighboring villages, and therefore not identified as a cluster in the statistical approach. To increase the accuracy of identifying clusters, especially in the urban areas, the program manager can consider using a different DBSCAN setting for urban areas or a different statistical approach that corrects for population densities. As a result, the proportion of cases in clusters may increase for both approaches. The drawback of increasing the proportion of cases in clusters is the more individuals need to be targeted for PEP and therefore more resources are needed.

### Strengths and limitations

Our contextualized spatial approach is a first step towards a feasible spatial approach that accounts for the local context. This approach has worked well in our study setting, and could therefore, be considered a useful method for program managers and policy makers if time and resources can be made available for collecting coordinates at individual level and contextual information. In addition, the output of the expert consultation would likely be relevant for the coming years as the local barriers don’t change much over time. The return of investment in time and resources can be significant because changes in, for example, the distribution of new leprosy cases in the studied areas, would require minimal updates to this approach. Further research in different settings is recommended to i) test and, if necessary, adapt the approach, ii) scale up to high-endemic leprosy districts, iii) apply to other neglected tropical diseases such as Buruli Ulcer, and iv) measure the cost-effectiveness of both approaches.

The DBSCAN clustering tool used in the contextualized spatial approach differs from other clustering algorithms by i) not using a pre-set number of clusters such as in the K-Means tool, but instead using a selected *Minpt* and maximum distance [31], and ii) not correcting for population densities. Selecting a maximum distance demands a proper understanding of the leprosy transmission and the population at risk in the study area. This was discussed in the expert consultation and a decision was made on the maximum distance for Chandauli and Fatehpur. A limitation in our study, however, is that we didn’t differentiate between rural and urban settings or correct for population densities and used the same DBSCAN parameters (*Minpt* of 2 and maximum distance of 500 m). For urban settings with a higher population density, program managers can consider selecting a higher *Minpt* and smaller maximum distance for the DBSCAN.

In the statistical spatial approach, we used the total number of cases as the event while other leprosy studies selected new case detection rate (NCDR) as event to identify clusters [17,18]. As a result, a village with a high number of cases but low NCDR due to large population can be identified as a cluster, while a village with a low number of cases but high NCDR due to a small population can be missed as a cluster. If we used the NCDR as the event, however, we would have included many rural villages with one leprosy case in both Chandauli and Fatehpur. Even though the NCDR is often used as measure of risk, a single leprosy case in a rural village may also be a sporadic case.

It should be noted that the use of spatial analysis to guide intervention targets and efficient use of public health resources is challenging, especially in high endemic districts. Clustering of leprosy cases can only be measured if the locations of registered cases are known. Cases that are not in the national health registers or from which the GPS coordinates are not collected are automatically excluded from the analysis. Such ‘hidden cases’ may be due to poor performance or absence of leprosy services at the health center level. These areas where hidden cases may exist will not be identified as high risk areas in the clustering analysis and not be targeted with (contact) screening interventions. While in these areas, public health resources may be used in an efficient way: detecting as many new cases of leprosy as possible while screening few individuals as possible. If a statistical approach is used, these areas will be identified as non-significant and will not be of interest for a program manager to target interventions resulting in continuous transmission of *M*. *leprae*. If a contextualized spatial approach is used, however, these results and areas will be discussed during the expert consultation and can be explained according to the local context or history. If it is expected that hidden cases may exist in these areas, the program manager can decide to implement or increase active case finding efforts. This can be followed by door-to-door PEP interventions or other PEP strategies to target the at risk populations and interrupt the transmission of *M*. *leprae*.

## Conclusion

This study showed that our contextualized spatial approach could identify clusters in high-endemic districts more precisely than a statistical spatial approach. It captures a larger proportion of cases in the identified clusters and covers a larger proportion of the population in the clusters (at risk) that would need to be targeted for a door-to-door approach with active case finding and PEP to support interruption of transmission of *M*. *leprae*. Therefore, this approach appears to be a useful approach to detect at-risk populations for preventive interventions in high-endemic areas. In other endemic settings, further research is needed to test the scalability.

## Supporting information

S1 Text Text document containing 1) the contextualized spatial approach, 2) number of new patients registered in the five mapping surveys conducted from January 2018 until June 2020 in Chandauli and Fatehpur (Table A), 3) block-wise epidemiological status of leprosy during the year 2020 to 2021 reported by the District Leprosy Office in Chandauli and Fatehpur (Table B), 4) the new case detection rate per 1,000,000 population per village for Chandauli and Fatehpur (Fig A), 5) heatmaps of Chandauli and Fatehpur district using a 100 m, 500 m, 1000 m, 2000 m and 3000 m radius (Fig B), and 6) DBSCAN clustering results of preliminary analysis (2014–2017) (Table C).
(DOCX)

## References

[1] Fischer M. Leprosy–an overview of clinical features, diagnosis, and treatment. JDDG—J Ger Soc Dermatology. 2017;15(8):801–27. Available from: https://onlinelibrary.wiley.com/doi/full/10.1111/ddg.13301 10.1111/ddg.13301 28763601

[2] Walker SL, Lockwood DNJ. The clinical and immunological features of leprosy. British Medical Bulletin. 2006;77–78(1):103–121. doi: 10.1093/bmb/ldl010 17090777

[3] Van Brakel WH, Sihombing B, Djarir H, Beise K, Kusumawardhani L, Yulihane R, et al. Disability in people affected by leprosy: the role of impairment, activity, social participation, stigma and discrimination. Global Health Action. 2012;5(18394). doi: 10.3402/gha.v5i0.18394 22826694 PMC3402069

[4] World Health Organization. Global leprosy (Hansen disease) update, 2019: time to step-up prevention initiatives Leprosy. Weekly Epidemiological Record. 2020;95(36):417–440.

[5] National Leprosy Eradication Programme (NLEP). Operational guidelines for leprosy case detection campaigns. 2016. Retrieved from https://pdfslide.net/reader/f/operational-guidelines-for-leprosy-case-detection-1pdf-draft-i-national-leprosy

[6] National Leprosy Eradication Programme (NLEP). Annual Report 2019–20. New Delhi. 2020. Retrieved from https://main.mohfw.gov.in/sites/default/files/Annual Report 2019–2020 English.pdf

[7] Rao P, Suneetha S. Current situation of leprosy in India and its future implications. Indian Dermatology Online Journal. 2018;9(2):83–89. doi: 10.4103/idoj.IDOJ_282_17 29644191 PMC5885632

[8] Towards Zero Leprosy. Global Leprosy (Hansen’s disease) Strategy 2021–2030. World Health Organization. New Delhi: World Health Organization, Regional Office for South-East Asia.2017. License: CC BY-NCSA 3.0 IGO

[9] Schoenmakers A, Mieras L, Budiawan T, Van Brakel WH. The state of affairs in post-exposure leprosy prevention: a descriptive meta-analysis on immuno-and chemo-prophylaxis. Research and Reports in Tropical Medicine. 2020;11:97–117. doi: 10.2147/RRTM.S190300 33117053 PMC7573302

[10] Moet FJ, Pahan D, Oskam L, Richardus JH; COLEP Study Group. Effectiveness of single dose rifampicin in preventing leprosy in close contacts of patients with newly diagnosed leprosy: cluster randomised controlled trial. BMJ (Clinical Research Ed.). 2008;336(7647):761–764. doi: 10.1136/bmj.39500.885752.BE 18332051 PMC2287265

[11] Guidelines for the diagnosis, treatment and prevention of leprosy. New Delhi: World Health Organization, Regional Office for South-East Asia. 2017. Licence: CC BY-NC-SA 3.0 IGO

[12] Auchincloss AH, Gebreab SY, Mair C, Diez Roux AV. A review of spatial methods in epidemiology, 2000–2010. Annu. Rev. Public Health. 2012;33:107–22. doi: 10.1146/annurev-publhealth-031811-124655 22429160 PMC3638991

[13] Bakker MI, Scheelbeek PFD, Van Beers SM. The use of GIS in leprosy control. Lepr Rev. 2009;80:327–31. Available from: https://www.lepra.org.uk/platforms/lepra/files/lr/sept09/1532.pdf 19961106

[14] Queiroz JW, Dias GH, Nobre ML, De Sousa Dias MC, Araújo SF, Barbosa JD, et al. Geographic Information Systems and applied spatial statistics are efficient tools to study Hansen’s disease (leprosy) and to determine areas of greater risk of disease. Am J Trop Med Hyg. 2010;82(2):306–14. doi: 10.4269/ajtmh.2010.08-0675 20134009 PMC2813173

[15] Albuquerque RA, Silva VMJ, Barreto EO, Alberto CFC, Oliveira dos Santos W, Salésia Moreira da Silva M, et al. Epidemiological, temporal and spatial dynamics of leprosy in a municipality in northeastern Brazil (2008–2017): an ecological study. Journal of the Brazilian Society of Tropical Medicine. 2020;53. 10.1590/0037-8682-0246-2020 PMC7580284 33111909

[16] Cury MRCO Paschoa, VD Nardi SMT, Chierotti AP Rodrigues Júnior AL, Chiaravalloti-Neto F. Spatial analysis of leprosy incidence and associated socioeconomic factors. Rev Saúde Pública. 2012;46(1):10–118. 10.1590/S0034-89102011005000086 22183514

[17] Daniel OJ, Adejumo OA, Oritogun KS, Kuye J, Akang G. Spatial distribution of leprosy in Nigeria. Lepr Rev. 2016;87(4):476–85. 10.47276/lr.87.4.476 30226352

[18] Dias Monteiro L, Rogerlândio Martins-Melo F, Brito AL, Alencar CH, Heukelbach J. Spatial patterns of leprosy in a hyperendemic state in Northern Brazil. Rev Saúde Pública. 2015;49(84):1–8. 10.1590/S0034-8910.2015049005866 PMC4650934 26603352

[19] De Assis IS, Berra TZ, Alves LS, Ramos ACV, Arroyo LH, Dos Santos DT, et al. Leprosy in urban space, areas of risk for disability and worsening of this health condition in Foz Do Iguaçu, the border region between Brazil, Paraguay and Argentina. BMC Public Health. 2020;20(1):119. 10.1186/s12889-020-8236-5 31996183 PMC6988226

[20] Kulldorff M. A spatial scan statistic. Communications in Statistics—Theory and Methods. 1997;26(6): 1481–1496. 10.1080/03610929708831995

[21] Kulldorff M, Heffernan R, Hartman J, Assunção R, Mostashari F. A space-time permutation scan statistic for disease outbreak detection. PLoS Medicine. 2005;2(3):0216–0224. doi: 10.1371/journal.pmed.0020059 15719066 PMC548793

[22] Bulstra CA, Blok DJ, Alam K, Butlin CR, Roy JC, Bowers B, et al. Geospatial epidemiology of leprosy in northwest Bangladesh: a 20—year retrospective observational study. Infectious Diseases of Poverty. 2021;10(36):1–12. doi: 10.1186/s40249-021-00817-4 33752751 PMC7986508

[23] Kuruwa S, Joshua V, Shetty V, Mistry N. Trends and spatial clustering of leprosy cases over a decade in a hyper-endemic area of western Maharashtra, India. Lepr Rev. 2016;87:294–304.

[24] Ramos ACV, Yamamura M, Arroyo LH, Popolin MP, Chiaravalloti Neto F, Palha PF, et al. Spatial clustering and local risk of leprosy in São Paulo, Brazil. PLoS Neglected Tropical Diseases. 2017;11(2):e0005381. 10.1371/journal.pntd.0005381 28241038 PMC5344525

[25] Neves KVRN, Nobre ML, Machado LMG, Steinmann P, Ignotti E. Misdiagnosis of leprosy in Brazil in the period 2003–2017: spatial pattern and associated factors. Acta Tropica. 2021;215:105791. doi: 10.1016/j.actatropica.2020.105791 33310076

[26] Taal AT, Blok DJ, Handito A, Wibowo S, Sumarsono, Wardana A, et al. Determining target populations for leprosy prophylactic interventions: a hotspot analysis in Indonesia. BMC Infect Dis. 2022;22(1):1–12. Available from: 10.1186/s12879-022-07103-0 35130867 PMC8822733

[27] Wu B. Embedding research in local context: local knowledge, stakeholders’ participation and fieldwork design [Internet]. 2014 [cited 2022 Jan 31]. Available from: https://blogs.lse.ac.uk/fieldresearch/2014/10/30/embedding-research-in-local-context.

[28] Suresh B. Role of GIS in planning and evaluation of Leprosy Elimination Programme. 2009 [cited 2021 Dec 15]. Published at Geospatial World.net. Available from: https://www.geospatialworld.net/article/role-of-gis-in-planning-and-evaluation-of-leprosy-elimination-programme/.

[29] Director General of Health Services-Ministry of Health & Family Welfare, Government of India; National Leprosy Eradication Program. State wise report 2019–20. [cited on 2021 Aug 12]. Availble from: https://dghs.gov.in/WriteReadData/userfiles/file/Leprosy/State%20wise%20report-2019-20.pdf

[30] Moran P. Notes on continuous stochastic phenomena. Biometrika. 1950;37(1):17–23. 15420245

[31] Ester M, Kriegel H-P, Sander J, Xu X. A density-based clustering methods for discovering clusters in large spatial databases with noise. Proc Second ACM Int Conf Knowl Discov Data Min. 1996;2:226–31.

[32] Anselin L. Local Indicators of Spatial Association-LISA. Geogr Anal. 1995;27(2):93–115.

[33] Meiyappan P, Roy PS, Soliman A, Li T, Mondal P, Wang S, et al. India village-level geospatial socio-economic data set: 1991, 2001. 2018 Palisades, NY: NASA Socioeconomic Data and Applications Center (SEDAC). [cited on 2021 Nov 11]. Available from: 10.7927/H4CN71ZJ.

[34] Meiyappan P, Roy PS, Sharma Y, Ramachandran RM, Joshi PK, DeFries RS et al. Dynamics and determinants of land change in India: integrating satellite data with village socioeconomics. Regional Environmental Change. 2017;17(3):753–66. doi: 10.1007/s10113-016-1068-2 32214900 PMC7064035

[35] World Health Organization. Global consultation of National Leprosy Programme managers, partners and affected persons on Global Leprosy Strategy 2021–2030: Report of the virtual meeting 26–30 October 2020. [cited on 2021 Dec 15]. Available from: https://www.who.int/publications-detail-redirect/9789290228226

[36] Jaeggi T, Manickam P, Weiss MG, Gupte MD. Stakeholders perspectives on perceived needs and priorities for leprosy control and care, Tamil Nadu, India. Indian J Lepr. 2012;84:177–184. 23484332

[37] Mieras LF, Taal AT, van Brakel WH, Cambau E, Saunderson PR, Smith WCS, et al. An enhanced regimen as post-exposure chemoprophylaxis for leprosy: PEP++. BMC Infectious Diseases. 2018;18(1):1–8. 30290790 10.1186/s12879-018-3402-4 PMC6173927

[38] Steinmann P, Dusenbury C, Addiss D, Mirza F, Smith WCS. A comprehensive research agenda for zero leprosy. Infectious Diseases of Poverty. 2020;9(1):1–7. 33183339 10.1186/s40249-020-00774-4 PMC7658911

[39] Fischer EAJ, Pahan D, Chowdhury SK, Richardus JH. The spatial distribution of leprosy cases during 15 years of a leprosy control program in Bangladesh: An observational study. BMC Infect. 2008;8(1):1–10. doi: 10.1186/1471-2334-8-126 18811971 PMC2564934

[40] Hoeven TA, Fischer EAJ, Pahan D, Richardus JH. Social distance and spatial distance are not the same, observations on the use of GIS in leprosy epidemiology. Epidemiol. Infect. 2008;136:1624–1627. doi: 10.1017/S0950268808000381 18272012 PMC2870776

[41] Mohite R V., Mohite VR, Durgawale PM. Differential trend of leprosy in rural and urban area of Western Maharashtra. Indian J Lepr. 2013;85(1):11–8. 24046910

[42] Cabral-Miranda W, Chiaravalloti Neto F, Barrozo LV. Socio-economic and environmental effects influencing the development of leprosy in Bahia, north-eastern Brazil. Trop Med Int Heal. 2014;9(12):1504–14. doi: 10.1111/tmi.12389 25244417

[43] Paschoal JAA, Paschoal VDA, Nardi SMT, Rosa PS, Ismael MGYS & Sichieri EP. Identification of urban leprosy clusters. Sci World J. 2013;2013:6. doi: 10.1155/2013/219143 24288467 PMC3833060

