# Supplementary material for: Identifying clusters of leprosy patients in India: A comparison of methods
Source: PLoS Negl Trop Dis. 2022 Dec 16;16(12):e0010972. doi: 10.1371/journal.pntd.0010972 (PMC9757546; doi:10.1371/journal.pntd.0010972)
Supplement: S1 Text — Text document containing 1) the contextualized spatial approach, 2) number of new patients registered in the five mapping surveys conducted from January 2018 until June 2020 in Chandauli and Fatehpur (Table A), 3) block-wise epidemiological status of leprosy during the year 2020 to 2021 reported by the District Leprosy Office in Chandauli and Fatehpur (Table B), 4) the new case detection rate per 1,000,000 population per village for Chandauli and Fatehpur (Fig A), 5) heatmaps of Chandauli and Fatehpur district using a 100 m, 500 m, 1000 m, 2000 m and 3000 m radius (Fig B), and 6) DBSCAN clustering results of preliminary analysis (2014–2017) (Table C). (DOCX) [file pntd.0010972.s001.docx]

S1 Text Contextualized spatial approach

**Data collection**

We conducted five mapping surveys between January 2018 and June 2020 to collect the GIS data for the PEP++ trial. Since leprosy registration in India is paper based recorded at the Primary Health Centers (PHCs), we obtained the patient’ names and addresses with village name from the medical records of all leprosy patients registered from April 2014 to March 2020 across 10 PHCs and 17 PHCs in Chandauli and Fatehpur, respectively. In January 2018, local PEP++ staff were trained in data collection using the mobile application MapIt (version 7.6.0, https://mapitgis.com/), a tool for Geographic Positioning Systems (GPS) data collection and management. They were assigned to one of the blocks and worked together with local government staff, such as Medical Officers (MOs), Non-Medical Assistants (NMAs), Paramedical workers (PMWs) of the PHC and Accredited Social Health Activists (ASHAs) as community volunteers. Since we only had the village and patient name, the support of local staff and AHSAs including the chief of village (i.e., *pradhans*) was required to find the patient’ house. Once the patient’s house was located, the GPS coordinates of the house were taken offline with MapIt. All data points collected during the five surveys were uploaded to the server in the NLR India office in New Delhi for aggregation combined with demographic information for analysis. Table A shows an overview of the five mapping surveys. All data were imported to the open-source Quantum Geographic Information System (QGIS) version 3.4.1 (QGIS Developer team, Madeira (2018)) for validation. Incorrect data points (e.g., situated in lake, field) were removed and recollected by local project staff. Population data was collected from the District Leprosy Office of both districts (Table B).

**Table A. Number of new patients registered of the five mapping surveys conducted from January 2018 until June 2020 in Chandauli and Fatehpur.**

|  | **1^st^ Survey** | **2^nd^ Survey** | **3^rd^ Survey** | **4^th^ Survey** | **5^th^ Survey** | **Total** |
| --- | --- | --- | --- | --- | --- | --- |
| **Date mapping** | Jan 2018 | April 2018 | Feb 2019 | May 2019 | June 2020 |  |
| **Time period** | April 2014-March 2017 | April 2017-March 2018 | April 2018-Dec 2018 | Jan 2019-March 2019 | April 2019-March 2020 |  |
| **Registered leprosy patients in Chandauli** | 981 | 209 | 269 | 33 | 208 | 1710 |
| **Total mapped** | 930 | 207 | 269 | 33 | 208 | 1647 |
| **Registered leprosy patients in Fatehpur** | 1091 | 367 | 412 | 106 | 373 | 2329 |
| **Total mapped** | 983 | 354 | 406 | 102 | 363 | 2208 |

**Table B. Block-wise epidemiological status of leprosy during the year 2020 to 2021 reported by the District Leprosy Office in Chandauli and Fatehpur. during the year 2020 to 2021 reported by the District Leprosy Office in Chandauli and Fatehpur.**

| **Chandauli** ^a^ | | | **Fatehpur** ^b^ | | |
| --- | --- | --- | --- | --- | --- |
| Block | Population (thousands)^c^ | NCDR (100,000)^d^ | Block | Population (thousands)^c^ | NCDR (100,000)^d^ |
| 1 | 267 | 7.49 | 1 | 241 | 4.99 |
| 2 | 329 | 4.26 | 2 | 208 | 8.64 |
| 3 | 176 | 7.97 | 3 | 232 | 17.7 |
| 4 | 227 | 5.28 | 4 | 224 | 11.19 |
| 5 | 108 | 5.55 | 5 | 174 | 7.45 |
| 6 | 314 | 4.15 | 6 | 197 | 5.07 |
| 7 | 250 | 6.39 | 7 | 206 | 7.78 |
| 8 | 240 | 9.18 | 8 | 253 | 12.23 |
| 9 | 269 | 2.97 | 9 | 217 | 5.06 |
| 10 | 166 | 1.81 | 10 | 202 | 1.48 |
|  |  |  | 11 | 231 | 9.52 |
|  |  |  | 12 | 233 | 3.42 |
|  |  |  | 13 | 226 | 3.98 |
|  |  |  | 14 | 202 | 8.93 |
|  |  |  | 15 | 61 | 1.65 |
| **Total** | **2346** | **5.46** |  | **3104** | **7.66** |

^a^ Data from District Leprosy Office Chandauli. Block Wise Epidemiological Status of Leprosy as S I S indicator during the year 2020-21. March 2021

^b^ Data from District Leprosy Office Fatehpur. Block Wise Epidemiological Status of Leprosy as S I S indicator during the year 2020-21. March 2021

^c^ Population as of 11-03-2020.

^d^ New Case Detection Rate (NCDR) is calculated as the number of new cases detected during the year 2020-21 divided by the population and multiplied by 100,000.

**Contextualized spatial approach**

We developed a contextualized spatial approach for the PEP++ trial to identify clusters of leprosy cases that account for the local barriers and social determinants. It comprises of the non-statistical density-based spatial clustering of applications with noise (DBSCAN) tool and an expert consultation to identify clusters of leprosy cases (high-transmission areas) at individual level. The approach was divided into three steps: a preliminary spatial analysis, an expert consultation to decide on criteria and a cluster definition, and the development of context specific cluster maps that can be used in the field. Fig 2 in the main text shows an overview of the steps.

***Step 1 Preliminary spatial analysis***

We analyzed the data collected in the first mapping survey (April 2014 to March 2017) on strength of clustering, then visualized clusters, and finally identified cases that were part of each main cluster. The data was analyzed using QGIS and GeoDa (<https://geodacenter.github.io/>) version 1.18 (Anselin, Santa Barbara, CA, USA). First, the new case detection rate per 1,000,000 population per village was calculated to identify high endemic villages for both Chandauli and Fatehpur (Fig A). Then, the strength of clustering of leprosy patients was calculated using Global Moran’s Index statistic [30] in GeoDa. It calculates the autocorrelation coefficient (degree of similarity) between spatial points (and takes values ranging between -1 to 1), where -1 indicates dispersed distribution, 0 no clustering, and 1 strong clustering. In this approach, the Global Moran’s I was used to determine reasonable cluster sizes. Strong clustering indicates that spatial points are located close together and therefore a cluster can contain many patients. Weak clustering indicates that only a few spatial points are close together and therefore a cluster should contain a few patients. We calculated a Global Moran’s I value of 0.06 (z score of 4.10 and p value of 0.002) for Chandauli and 0.04 (z score of 3.83 and p value of 0.005) for Fatehpur indicating weak clustering of leprosy cases.


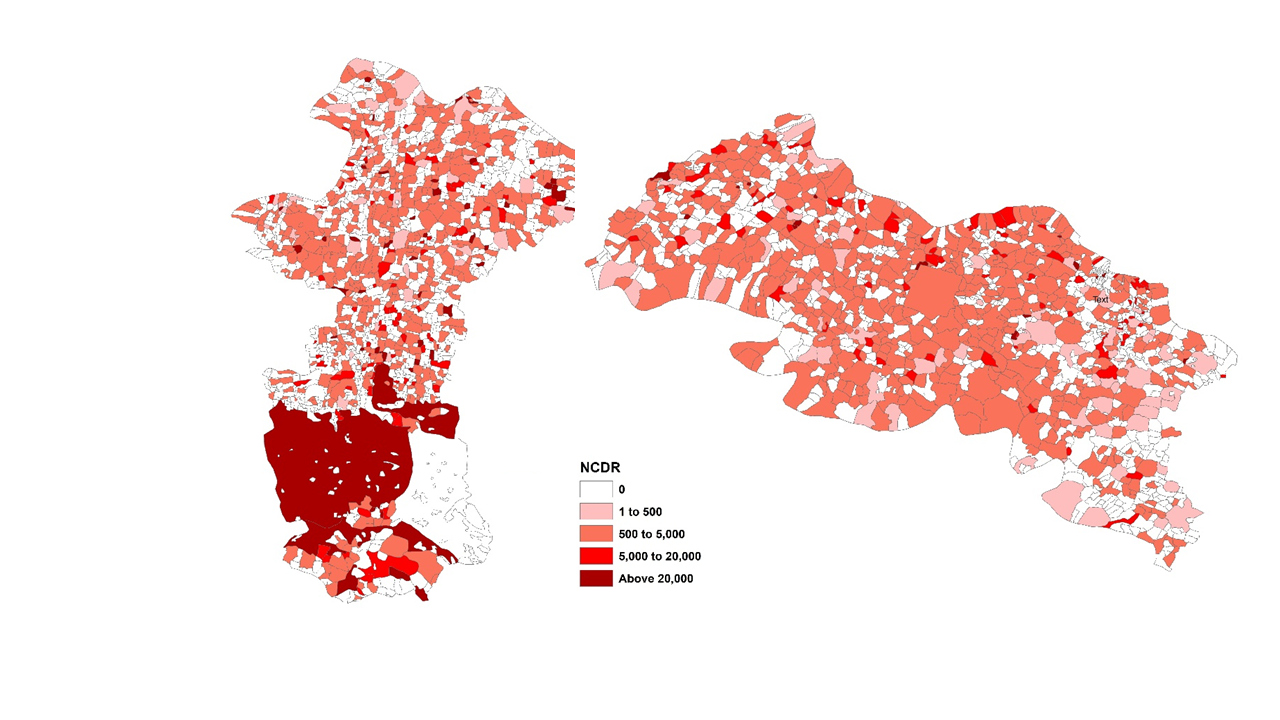


**Fig A. The new case detection rate per 1,000,000 population per village for Chandauli (left) and Fatehpur (right). Base layer from NASA Socioeconomic Data and Applications Center (SEDAC): https://sedac.ciesin.columbia.edu/data/set/india-india-village-level-geospatial-socio-econ-1991-2001/data-download.**

Clustering of cases was visualized using the heatmap tool in QGIS (Heatmap plugin). The heatmap tool draws a circle with a specified radius (e.g., 1000 m) around each data point and creates a raster file as output. Then, it uses Kernel Density Estimation to calculate the density of points for each raster cell. Raster cells that are close to data points will have a higher density (higher value) compared to raster cells that are further away which will have a low density (low value). The resulting map showed the density distribution of clusters and non-clusters. Fig B shows the density distribution of clusters and non-clusters using five different radii (100 m, 500 m, 1000 m, 2000 m, 3000 m) to visualize clustering of leprosy cases in Chandauli and Fatehpur. In this approach, the heatmap radius was used to select the maximum distance between patients that would be part of a cluster. A heatmap that shows many individual density spots can be selected as the maximum distance.


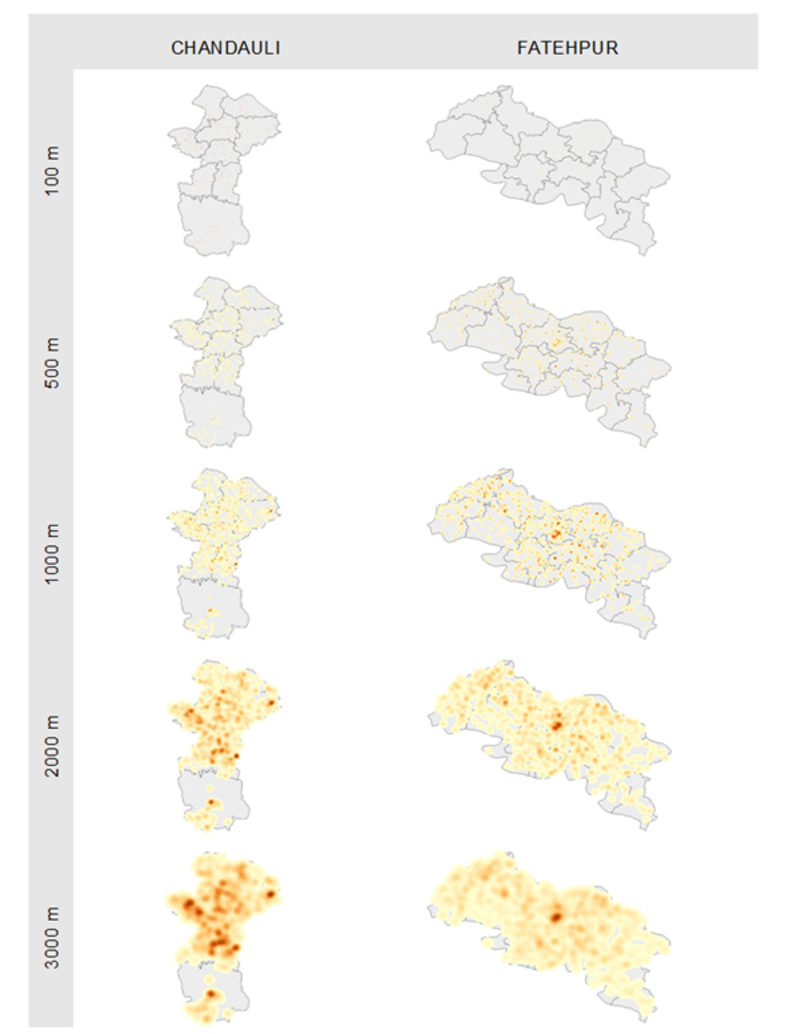


**Fig B. Heatmaps (density distribution) of Chandauli (left) and Fatehpur district (right) using a 100 m, 500 m, 1000 m, 2000 m and 3000 m radius and the singleband pseudocolor yellow (low density) to dark brown (very density). Base layer from NASA Socioeconomic Data and Applications Center (SEDAC): https://sedac.ciesin.columbia.edu/data/set/india-india-village-level-geospatial-socio-econ-1991-2001/data-download.**

To identify cases that are part of a cluster, we used the DBSCAN tool [31] of QGIS. This tool focuses on the proximity and density of points to form (arbitrary shaped) clusters. A minimum cluster size (minimum points; *MinPts*) and maximum distance to a nearest neighbor (radius; ε) needs to be selected beforehand. A point is identified as a cluster point if it has the specified minimum number of points in its radius-neighborhood. The parameters of the DBSCAN were determined by the strength of clustering (Global Moran’s I) and density of points (Heatmap). We used the DBSCAN tool with 12 different combinations of minimum cluster size (i.e., minimum points = 2, 3, 4) and maximum distance (i.e., radius = 200 m, 300 m, 400 m, and 500 m). For each combination, we calculated the proportion of total cases georeferenced that would be part of a cluster (Table C).

**Table C. DBSCAN clustering results of preliminary analysis (2014-2017).**

|  | | Chandauli | | | Fatehpur | | |
| --- | --- | --- | --- | --- | --- | --- | --- |
| Cluster setting | | Number of cases in clusters | Proportion of cases in clusters | Number of clusters | Number of cases in clusters | Proportion of cases in clusters | Number of clusters |
| Cluster size | Distance in m |  |  |  |  |  |  |
| 2 | **200** | 446 | 48% | 154 | 421 | 43% | 174 |
|  | **300** | 533 | 57% | 178 | 514 | 52% | 201 |
|  | **400** | 586 | 63% | 187 | 567 | 58% | 203 |
|  | **500** | 640 | 69% | 197 | 603 | 61% | 206 |
| 3 | **200** | 262 | 28% | 62 | 177 | 18% | 52 |
|  | **300** | 337 | 36% | 80 | 258 | 26% | 73 |
|  | **400** | 388 | 42% | 88 | 335 | 34% | 87 |
|  | **500** | 438 | 47% | 96 | 365 | 37% | 87 |
| 4 | **200** | 164 | 18% | 30 | 52 | 5% | 11 |
|  | **300** | 211 | 23% | 39 | 107 | 11% | 23 |
|  | **400** | 257 | 28% | 45 | 192 | 20% | 41 |
|  | **500** | 297 | 32% | 51 | 236 | 24% | 45 |

***Step 2: Expert consultation***

In February 2018, a 3-day expert consultation was organized in New Delhi (day 1 and 2) and Lucknow (day 3), India. The aim of the expert consultation was i) to discuss the preliminary results of the clustering analysis (day 1 and 3), ii) to decide on cluster definition and DBSCAN setting (day 2 and 3), and iii) to discuss the implications of this choice for the door-to-door campaigns in both districts (day 2 and 3). Among the participants were the Deputy Director General of the National Leprosy Elimination Program of India, representatives of the UP state and district government health department of Chandauli and Fatehpur, local health care workers, ASHAs, NLR branch and foundation staff, and PEP++ staff (n=60). Their knowledge and experience of the Chandauli and Fatehpur context were important to discuss the results and define a cluster.

To get a better understanding of the Chandauli and Fatehpur context, relevant questions were asked related to the i) transmission of *M. leprae* in the two districts, including MB leprosy, child, and grade 2 disability proportions, ii) the travel and social behavior of leprosy infected persons, and iii) specifications of the environment (i.e., districts), including rural or urban areas, natural and social boundaries, population counts, poverty, presence of violence or stigma and access to health centers.

The Global Moran’s I and the five constructed heatmaps for each district were discussed by the experts. They recommended using a small cluster size (*Minpt*) considering weak clustering. Moreover, a small radius of 500 m showed many individual low density spots and only a few medium density spots, while a large radius of 3000 m showed a smoothen high density spot covering the whole district (Fig B). Considering that the PEP++ trial has limited resources and aims to target only the high transmission areas (clusters) with PEP door-to-door campaigns, it was recommended using a small radius for further cluster analysis. A large radius would have resulted in targeting the complete district.

In this study, the experts chose to determine a leprosy cluster based on a proportion of cases in clusters. This proportion can be decided in different ways, for example trial data or what is feasible for the studied area (pragmatic). Here, we used preliminary modelling predictions specifically made for the PEP++ study (not published). It was shown that if 50% of the population in villages with leprosy cases were targeted with SDR-PEP significant reductions in NCD could be achieved in five years. Considering a 10% margin, we therefore determined that at least 60% of the leprosy cases should be part of a detected cluster. The experts estimated that on average 100 individuals (or 20 households) per leprosy cluster case should be targeted to achieve this coverage.

The DBSCAN preliminary results were discussed and tested on the criterion. The combination that resulted in at least 60% of the cases in a cluster was a minimum size of a cluster of 2 and maximum distance of 500 m (Table C). Other combinations had a smaller proportion of cases in clusters. The experts decided that the definition of a cluster for the two districts should be ‘at least 2 cases living within 500 m of each other’.

***Step 3: Develop context specific cluster maps***

The data points that are within 500 m of another point were identified as a cluster case and separated from the dataset. Cases that are part of the same cluster were grouped and we drew a polygon shape around each group using Google Satellite Imagery. While shaping the areas, we considered the village, street and natural boundaries, and a buffer zone that contains on average 100 individuals (i.e., 20 households) per cluster case as was decided during the expert consultation.

The cluster maps were updated after every mapping survey and discussed with the PEP++ staff and the *pradhans* in Chandauli and Fatehpur for validation. After mapping survey 5 (2014-2020), 374 clusters were identified in Chandauli and 512 clusters in Fatehpur. Based on the final cluster maps, the total population of each cluster was assessed by a field visit. The interaction with the gram Pradhan and community members has provided rough estimates of the cluster populations.

The final clusters were numbered and divided into small (2 to 3), medium (4 to 6) and large (7 to 9) clusters based on the number of cases in clusters. We developed pragmatic context specific maps for each cluster that can be used by the PEP++ staff during the door-to-door campaigns.
